# Indoor Positioning Based on Pedestrian Dead Reckoning and Magnetic Field Matching for Smartphones

**DOI:** 10.3390/s18124142

**Published:** 2018-11-26

**Authors:** Jian Kuang, Xiaoji Niu, Peng Zhang, Xingeng Chen

**Affiliations:** 1 GNSS Research Center, Wuhan University, No. 129 Luoyu Road, Wuhan 430079, China; kuang@whu.edu.cn (J.K.); xingengchen@whu.edu.cn (X.C.); 2 Collaborative Innovation Center of Geospatial Technology, Wuhan University, No. 129 Luoyu Road, Wuhan 430079, China; 3 State Key Laboratory of Information Engineering in Surveying, Mapping and Remote Sensing, Wuhan University, No. 129 Luoyu Road, Wuhan 430079, China; fenix@whu.edu.cn

**Keywords:** magnetic field, pedestrian navigation, indoor positioning, MM, INS, PDR

## Abstract

This paper presents an ambient magnetic field map-based matching (MM) positioning algorithm for smartphones in an indoor environment. To improve the low distinguishability of a magnetic field fingerprint at a single point, a magnetic field sequence (MFS) combined with the measured trajectory contour coming from pedestrian dead-reckoning (PDR) is used for MM. Based on the fast approximation of magnetic field gradient, a Gauss-Newton iterative (GNI) method is used to find a rigid transformation that optimally aligns the measured MFS with a reference MFS coming from the magnetic field map. Then, the position of the reference MFS is used to control the position drift error of the inertial navigation system (INS) based PDR by an extended Kalman filter (EKF) and to further improve the accuracy of the trajectory contour. Finally, we conduct several experiments to evaluate the navigation performance of the proposed MM algorithm. The test results show that the position estimation error of the MM algorithm is 0.64 m (RMS) in an office building environment, 1.87 m (RMS) in a typical lobby environment, and 2.34 m (RMS) in a shopping mall environment.

## 1. Introduction

Global navigation satellite systems (GNSSs) can provide accurate location service for pedestrians in open-sky outdoor scenarios, but GNSSs are hindered by signal attenuation and blockage in certain environments (e.g., indoor environments). Therefore, to provide ubiquitous location service, a number of technologies have been developed for indoor positioning, such as pseudo-satellites [1], ZigBee [2], ultra-wideband (UWB) [3], radio frequency identification (RFID) [4], infrared [5], ultrasonic [5], iBeacon [6]. These methods are all capable of providing a high-precision positioning service for pedestrians over a long time scale in an indoor environment. However, specialized provisioning and regular maintenance are necessary in order to sustain the performance of the system. Their costs are therefore always very high for a wide coverage area.

Smartphone-based Wi-Fi fingerprint localization technology has become the most popular indoor positioning method due to the worldwide availability of Wi-Fi access points (APs) and the unprecedented growth of smartphones with built-in Wi-Fi receivers. Unfortunately, it is still expensive to maintain a robust Wi-Fi-based localization system due to the unexpected changes in the position and working status of Wi-Fi APs [7,8].

Recently, many researchers have found that precise indoor positioning based on magnetic fingerprints will become possible due to the intense magnetic field interference and anomalies caused by the metal structure of the building [9]. Subbu et al. [10] analyzes the feasibility of indoor magnetic field-based positioning method from the aspects of building structure, smartphone types, the placement of a smartphone, the presence of a furniture and the presence of personal metallic objects. Compared with Wi-Fi fingerprints, magnetic field fingerprint-based positioning has many advantages: (a) magnetic fields are available everywhere and there is no deployment requirement for extra specialized infrastructure; (b) the distribution of the magnetic field is quite steady over a given time scale of several months [11,12,13,14], and the number of people in spaces have little effect on the magnetic field [15,16]; (c) magnetic field fingerprint-based positioning has better positioning performance than Wi-Fi fingerprints [17,18].

There have been increasing efforts in exploiting ambient magnetic-based indoor positioning methods for pedestrians using a smartphone [19]. Magnetic field sequence-based dynamic time warping (DTW) and single point magnetic field-based particle filter (PF) are the two typical methods. Subbu and Riehle et al. [20,21] describe the basic process of using DTW to calculate the similarity between the measured MFS and the candidate MFS coming from the magnetic field map. The follow-up research can be divided into three categories: (a) to eliminate the influence of device heterogeneity, the differential values of the measured and candidate MFS are used to do a comparison [22]; (b) based on the fact that the distribution of magnetic field varies with the spatial scale, these systems perform the DTW after resampling the raw magnetic field measurement according to the user’s moving distance given by a PDR [10]; (c) for the magnetic field fingerprint is not unique over a wide range of region [11,15], many other methods are used to reduce the search scope, such as Wi-Fi fingerprints [8], building structures (e.g., doors and corners) [13], and the pre-planned reference track when navigating [22]. The basic principle of DTW-based MM is revisiting the historical trajectory stored in the magnetic field map during the positioning phase. Thus, this algorithm will perform poorly in open indoor areas because the user’s walking trajectories cannot be predicted well during the magnetic field map training phase.

Different from DTW, PF keeps the basic framework of the filtering idea that individually processing a single point magnetic field measurement, and recursively re-samples a set of particles according to the results of comparing the measured magnetic fingerprint and the reference magnetic fingerprint to converge to the true position. Experiments in [23] show that the single point magnetic field-based particle filter has the ability to converge to the real position in a straight corridor environment. Grand et al. [16] presents an online particle filter that achieved localization accuracy of 0.7 m in a square room. Putta et al. [24] uses gradient descent algorithm to improve users’ heading estimation, and utilizes magnetic field maps and indoor maps to improve the particles weight estimation. Test results show that the system achieves a mean localization accuracy of 0.75 m. Kim et al. [25,26] propose magnetic gradient sequence based technique to mitigate the influence of the bias of magnetometer, which has much more adaptable to different user devices than the conventional PF. Xie et al. [27] applied an adaptive step length estimation algorithm, magnetic field gradient sequence and a heuristic particle resampling algorithm to PF, and the results showed that this system has an excellent robustness, convergence speed and positioning accuracy performance. As reported in [16,17,23,24,25,26,27,28,29,30], PF performs well in robustness, positioning accuracy, and ease of implementation. However, improper setting of motion estimation, particle weight update and resampling may lead to slow convergence and less robustness to external signal noise. Moreover, to ensure the positioning performance, the number of particles cannot be too small, so its large computational load is still not suitable for smartphones.

Inspired by the traditional magnetic field matching algorithms, we use magnetic field sequences combined with the trajectory contour to improve the positioning performance of a smartphone. The traditional algorithms mainly include the terrain contour matching (TERCOM) and the iterative closest contour point (ICCP). TERCOM holds on the idea that the measured trajectory from an inertial navigation system (i.e., INS) has an error in the initial position [31]. Thus, only the position translation is considered when calculating the similarity of the observed MFS and the reference MFS in the magnetic field map. In contrast to TERCOM, ICCP based on the idea that the initial position error and heading error have the same influence on the similarity calculation [32].

Due to the fact smartphones’ built-in micro-electromechanical-system (MEMS) IMU (consisting of a tri-gyroscope and a tri-accelerometer) are generally of poor quality, different from TERCOM and ICCP, we use a pedestrian dead reckoning (PDR) method instead of the pure strap down inertial navigation algorithm to generate the contour feature of a magnetic field sequence. The pedestrian-gait-model-based (i.e., gait-based) and inertial-navigation-system-based (i.e., INS-based) are the two typical PDR methods [33]. Gait-based PDR determines pedestrians’ position following four basic modules: step event detection, step length estimation, moving direction estimation and two-dimensional (2D) position calculation [34,35]. INS-based PDR propagates the users’ position by integrating the angular rate and specific force coming from the tri-gyroscope and tri-accelerometer, respectively [33,36,37,38]. This strategy uses the estimated step length to produce a moving velocity update and position increment update to improve the PDR navigation performance. Based on the test results of [33], INS-based PDR will be more suitable for providing the contour feature than gait-based since its higher output data rate and more robust navigation performance.

In this paper, we present an ambient magnetic field-based positioning method for smartphones indoors. To improve the low distinguishability of the magnetic field fingerprint at a single point, an MFS combined with the measured trajectory contour coming from an INS-based PDR is used for MM instead. Based on the fast approximation of magnetic field gradient, we employ a GNI method to find the reference sequence with the highest similarity to the measured sequence in the magnetic field map. Then, the position of the MM is used to control the navigation error accumulation of the INS by an extended Kalman filter (EKF) and improve the accuracy of the trajectory contour. Finally, we describe several experiments conducted to evaluate the positioning performance of the proposed algorithm.

This paper makes the following three main contributions:

- We employ a strap-down INS-based PDR to provide the relative trajectory contour for a MFS, which can improve the low distinguishability of the magnetic field fingerprint at a single point.
- We devise a novel online magnetic-field-fingerprint-based matching positioning method (e.g., Gauss-Newton iterative method). The proposed algorithm is suitable for smartphone because of its high positioning accuracy and low computational load.
- We implement the proposed method on Android smartphones and evaluate it in three buildings with 2 participants. The position errors are 0.64 m (RMS) in an office building environment, 1.87 m (RMS) in a typical lobby environment, and 2.34 m (RMS) in a shopping mall environment.

The remainder of the paper is organized as follows: Section 2 summarizes the indoor positioning solution process and gives a detailed description of each module, such as sensors calibration, INS mechanization, the designed EKF, measurement information updating, magnetic field map training, and the magnetic matching method; Section 3 investigates the positioning performance of the proposed magnetic matching method; Section 4 discusses the advantages and disadvantages of the proposed method; and Section 5 draws the conclusions.

## 2. Materials and Methods

**Figure 1:**
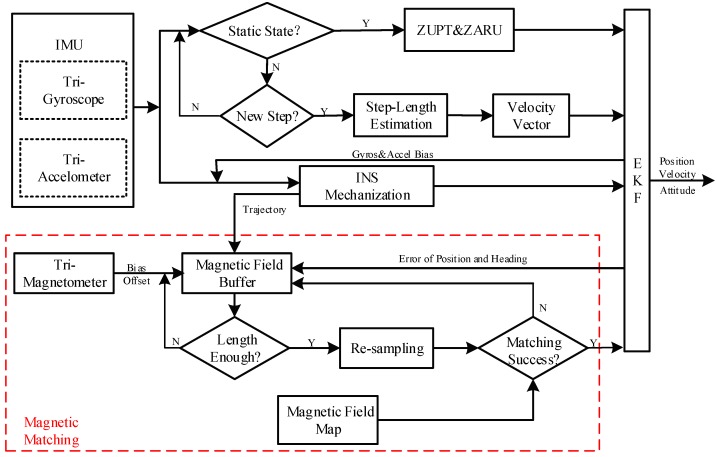
Detailed indoor positioning solution process.

Figure 1 shows our proposed indoor positioning method. The output of the IMU is utilized to set up the INS mechanization and detect the motion states (i.e., quasi-static and step detection). If the quasi-static state detection is successful, zero-velocity update technology (ZUPT) and a zero angular rate update (ZARU) are used as the velocity update and heading update, respectively, for the INS to control the velocity error and improve the heading estimation. However, if the quasi-static state detection fails and the step detection is successful, the step length estimation is executed to calculate the walking velocity of the pedestrians. The walking velocity functions as the velocity update for the INS to improve the velocity estimation. The tri-magnetometer measurements combine with the trajectory contour coming from INS are input into the data buffer to set up the MFS. If the buffered length of the magnetic field data exceeds the preset threshold, the raw MFS is resampled and a Gauss-Newton iterative method is used to find a reference sequence with the highest similarity to the measured sequence in the magnetic field map. If MM is successful, the position of the reference MFS functions as a position update for controlling the navigation error of the INS and improving the accuracy of the trajectory contour.

### 2.1. Sensors Calibration

Due to the inevitable presence of sensor error, the output of the tri-magnetometer will not reflect the real magnetic field of the surrounding environment. Therefore, it is necessary to perform the sensor calibration process before using a tri-magnetometer to perceive the magnetic field. 

**Figure 2:**
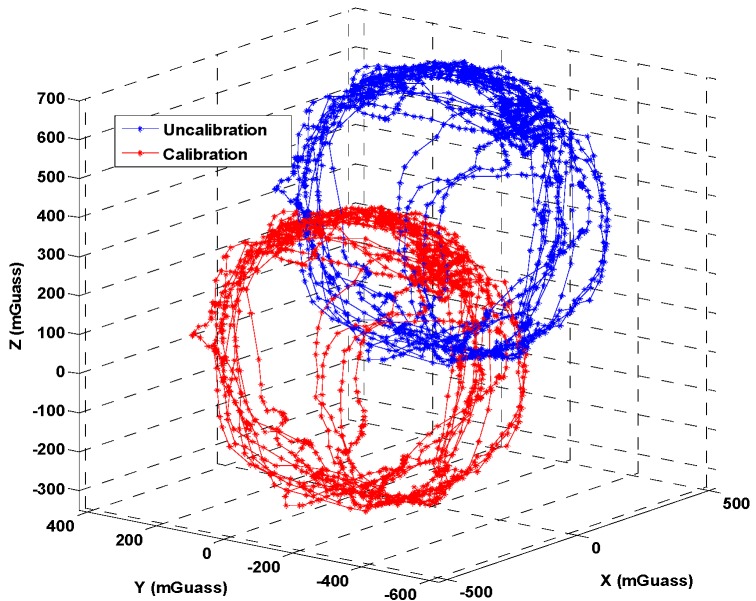
Magnetometer bias calibration.

In general, the sensor error of a tri-magnetometer can be divided into hard iron and soft iron effects [39,40]. The hard iron effect is only an additional magnetic field generated by permanent magnets or electrical currents, and the soft iron effect is caused by a material that produces its own magnetic field in intensity and direction. These materials respond to and distort the underlying magnetic field [41]. As the effect of the soft iron effect is small and common, we calibrate only the hard iron effect to simplify the calibration process. Figure 2 shows the calibration result of the Huawei P10 built-in tri-magnetometer, and a detailed calibration algorithm can be found in [42]. In addition, the bias of tri-gyroscope and tri-accelerometer will not be calibrated separately, as we will estimate them in the online positioning phase by using the designed algorithm.

### 2.2. INS-Based Navigation

As the magnetic field fingerprint has a maximum of only three components at one point, we utilize a PDR method to produce a relative trajectory contour to improve the discrimination of the magnetic field fingerprint. As the gait-based PDR has a typical output rate of 1–2 Hz in position, we use an INS-based PDR with a higher frequency output (e.g., 50 Hz) to provide a relative trajectory contour alternative [33].

#### 2.2.1. INS Mechanization

The basic idea of INS mechanization is that the position, velocity, and attitude of an object can be obtained by integrating the specific forces and angular rates provided by tri-accelerometers and the tri-gyroscope. Due to the low quality of smartphones’ built-in MEMS IMU, we have neglected certain small error correction terms (i.e., rotation of the Earth) of the INS mechanization for their slight improvement in navigation performance. Discrete INS mechanization algorithm equations can be presented in simplified form as follows [33]:

(1) $$\left(\begin{array}{c} r_{k}^{n}\\ v_{k}^{n}\\ C_{b,k}^{n} \end{array}\right)=\left(\begin{array}{c} r_{k-1}^{n}+v_{k}^{n}\Delta t\\ v_{k-1}^{n}+\left[C_{b,k}^{n}\left({\tilde{f}}_{k}^{b}-b_{a}\right)-g^{n}\right]\Delta t\\ C_{b,k-1}^{n}+C_{b,k-1}^{n}\Omega \left[\left({\tilde{\omega }}_{k}^{b}-b_{g}\right)\Delta t\right] \end{array}\right)$$

where $r^{n}$ represents the position vector in the navigation coordinate system frame (i.e., *n*-frame); $v^{n}$ is the velocity vector in the *n*-frame; $C_{b}^{n}$ is the transformation matrix from the body coordinate system (i.e., *b*-frame) to the *n*-frame as a function of attitude components; $g^{n}={\left[\begin{array}{ccc} 0 & 0 & -g \end{array}\right]}^{T}$ and is the Earth gravity vector in the *n*-frame; ${\tilde{f}}^{b}$ and ${\tilde{\omega }}^{b}$ are the acceleration and angle rate measurement vector, respectively; $b_{a}$ and $b_{g}$ are the bias vector of the tri-accelerometer and tri-gyroscope, respectively; $\Delta t=t_{k}-t_{k-1}$ is the time interval between the (*k* − 1)-th and *k*-th epochs; and Ω[•] is the cross-product form of a vector. 

#### 2.2.2. Extended Kalman Filter Design

Low-grade MEMS IMUs always have large acceleration and gyroscope biases, which will cause a rapid accumulation of positioning errors for a MEMS–IMU-based positioning system. To fix this issue, an EKF is usually used to fuse multi-source observations to improve the system availability and positioning accuracy. The state variables in the proposed method are defined as follows [33]:

(2) $$\delta x={\left[\begin{array}{ccccc} {\left(\delta r^{n}\right)}_{1\times 3} & {\left(\delta v^{n}\right)}_{1\times 3} & \psi_{1\times 3} & {\left(b_{g}\right)}_{1\times 3} & {\left(b_{a}\right)}_{1\times 3} \end{array}\right]}^{T}$$

where $\delta r^{n}$ represents the position error vector in the *n*-frame, $\delta v^{n}$ the velocity error vector in the *n*-frame, and ψ the attitude error vector in the *n*-frame. $b_{g}$,$b_{a}$ are the bias error vectors of the tri-gyroscope and tri-accelerometer, respectively. The discrete linearization of the system error model can be expressed as follows:

(3) $$\{\begin{array}{c} \delta x_{k|k-1}=\Phi_{k-1}\delta x_{k-1|k-1}+w_{k}\\ \delta z_{k}=H_{k}\delta x_{k|k-1}+v_{k} \end{array}$$

where the subscripts *k* − 1 and *k* represent the epoch, δz represents the measurement misclosure vector, H the design matrix, w the process noise, **v** the measurement noise, **Φ** the 15 × 15 state transition matrix can be found in the reference [33].

#### 2.2.3. Measurement Information Update

Abundant constraint information can be used as measurement information for improving the navigation performance of the INS, such as the gravity vector, quasi-static magnetic field vector, zero-velocity update technology (ZUPT), zero angular rate update (ZARU), and gait-model based velocity vector. The proposed algorithm considers that the basic pattern of human motion performs a stationary state in the absence of movement or repeated walking [43]. Thus, ZUPT and gait-model based velocity vector will be available.

To separate the “static” state and the “walking” state, the raw output of the tri-accelerometer and tri-gyroscope is employed to detect the stationary state period:

(4) $$\frac{1}{W}\sum_{k=1}^{W}(\frac{{\left\|\omega_{k}^{b}\right\|}^{2}}{\sigma_{\omega }^{2}}+\frac{1}{\sigma_{f}^{2}}\cdot \left\|f_{k}^{b}-g^{n}\cdot \frac{{\bar{f}}^{b}}{\left\|{\bar{f}}^{b}\right\|}\right\|)<\varepsilon$$

where W represents the window length of the stationary state detection, $\omega_{k}^{b}$ and $f_{k}^{b}$ are the vectors of the angular rate and the specific force at epoch *k*, respectively; ${\bar{f}}^{b}$ is the mean value vector of the specific force, $\sigma_{\omega }$ and $\sigma_{f}$ are the sensor noises of the gyros and accelerometer, respectively; and ε is the threshold of the stationary state determination. If the pedestrian is detected as the “static” state, the velocity should be zero. A velocity observation equation in the n-frame for ZUPT is given by:

(5) $$\delta z_{v}={\hat{v}}_{ins}^{n}-{\left[\begin{array}{ccc} 0 & 0 & 0 \end{array}\right]}^{T}=\delta v^{n}+n_{v}$$

where ${\hat{v}}_{ins}^{n}$ represents the velocity vector coming from the INS mechanization in the *n*-frame and $n_{v}$ is the measurement noise. Moreover, the change in heading should be zero. A heading observation equation in the *n*-frame for ZARU is given by:

(6) $$\delta z_{\psi }={\hat{\psi }}_{ins}-\psi_{store}=\left[\begin{array}{ccc} \frac{\partial \psi }{\partial \phi_{x}} & \frac{\partial \psi }{\partial \phi_{y}} & \frac{\partial \psi }{\partial \phi_{z}} \end{array}\right]\phi +n_{\psi }$$

where ${\hat{\psi }}_{ins}$ represents the heading coming from the INS mechanization, $\psi_{store}$ is the stored heading at the first epoch of the stationary state period, and $n_{\psi }$ the measurement noise. $\left[\begin{array}{ccc} \frac{\partial \psi }{\partial \phi_{x}} & \frac{\partial \psi }{\partial \phi_{y}} & \frac{\partial \psi }{\partial \phi_{z}} \end{array}\right]$ represents the heading design matrix, the detailed equation of which can be found in reference [44].

In the “walking” state, we can reasonably assume that there is speed only in the forward direction, while the velocity is zero in the lateral and vertical directions. The peak detection algorithm is employed to detect a step event for its lower computation, and the Weinberg model is utilized to estimate the step length due to less-estimated parameters [33]. After the step estimation is completed, the velocity vector in the *b*-frame can be expressed as follows:

(7) $${\tilde{v}}^{b}={\left[\begin{array}{ccc} SL/\Delta t & 0 & 0 \end{array}\right]}^{T}$$

where SL represents the step length. The computed velocity between the adjacent two steps in the *b*-frame can be expressed as:

(8) $${\hat{v}}^{b}\approx {\hat{C}}_{n}^{b}{\hat{v}}^{n}\approx v^{b}+C_{n}^{b}\delta v^{n}-C_{n}^{b}\left(v^{n}\times \right)\psi$$

and the velocity observation equation in the *b*-frame is given by:

(9) $$\delta z_{v}={\hat{v}}^{b}-{\tilde{v}}^{b}=C_{n}^{b}\delta v^{n}-C_{n}^{b}\left(v^{n}\times \right)\psi +n_{v}$$

### 2.3. Magnetic Field Matching Positioning

Magnetic field contour matching positioning mainly includes two phases: an offline training phase and an online positioning phase. In the offline training phase, a one-to-one correspondence between the geographic coordinates and the magnetic field fingerprints is established (i.e., a magnetic field map). In the online positioning phase, a sequence coming from the magnetic field map with the highest similarity to the measured sequence will be determined (i.e., matching positioning).

#### 2.3.1. Offline Training Phase

Although a magnetic field fingerprint has a maximum of three components at one point, only two components are available because the absolute heading is often unknown or inaccurate. We use the roll and pitch arising from INS mechanization to obtain the horizontal and vertical components of the magnetic field strength. The vertical magnetic intensity can be calculated as follows [8]:

(10) $$m_{v}=-\sin\theta \cdot m_{x}+\sin\phi \cos\theta \cdot m_{y}+\cos\phi \cos\theta \cdot m_{z}$$

where $m_{x},m_{y},m_{z}$ represent the raw measurement of tri-magnetometer, ϕ,θ are the roll and pitch, respectively; and $m_{v}$ is the vertical component of the magnetic intensity. The horizontal component of the magnetic intensity can be obtained as $m_{h}=\sqrt{{\left\|m\right\|}^{2}-m_{v}^{2}}$, where $\left\|m\right\|=\sqrt{m_{x}^{2}+m_{y}^{2}+m_{z}^{2}}$ represents the total magnetic intensity. Thus, the magnetic field fingerprint can be expressed as $M=\left[m_{h},m_{v}\right]$ at one point.

To ensure the positioning accuracy of MM, a high-precision magnetic field map is necessary. The coordinates of the reference points (RPs) and the observations of the magnetic field at the RPs are the two main factors affecting the accuracy of the magnetic field map. As per the description of Section 2.1, the error of the magnetic field observations from the tri-magnetometer can be ignored after the bias calibration process. Thus, the key is determining how to obtain accurate coordinates of the RPs to generate a high-precision magnetic field map.

Various methods have been designed for measuring the coordinates of RPs indoors. Standing still at each RP to measure the magnetic field strength is a typical method for generating a magnetic field map. First, the interested area is equally divided into a number of grids, and a survey method (e.g., total station) is used to measure the coordinates of the grids (i.e., the coordinates of the RPs). Second, the surveyors will stand at each RP for a few seconds to collect the magnetic field data. It is obvious that standing still at each RP cannot meet the need to produce a magnetic field map for a wide area due to its complex procedure and low efficiency. To fix this issue, the walking survey (WS) inspired by the method of standing still at each RP and PDR is proposed to improve the work efficiency of building a magnetic field map. The basic idea is to use the planning trajectories (consisting of many straight lines) to ensure the surveying density of the RPs. And to obtain high precision coordinates of the RPs, the sparse waypoints (i.e., points with known coordinates) and the constant walk velocity constraint are necessary [45].

In addition, an automatic robot with a tri-magnetometer can be utilized to train the magnetic field map [16]. As the robot uses the simultaneous localization and mapping (i.e., SLAM) to determine its own position without prior knowledge of the test environment and assistance from the other survey methods [46], it has excellent performance in providing the high-precision coordinates of the RPs. However, an automatic robot will cost approximately 20 times the price of a smartphone; thus, the cost of this method will grow dramatically when a large area must be covered at the same time. Furthermore, the electromagnetic field caused by the robot’s motor will distort the underlying magnetic field.

**Figure 3:**
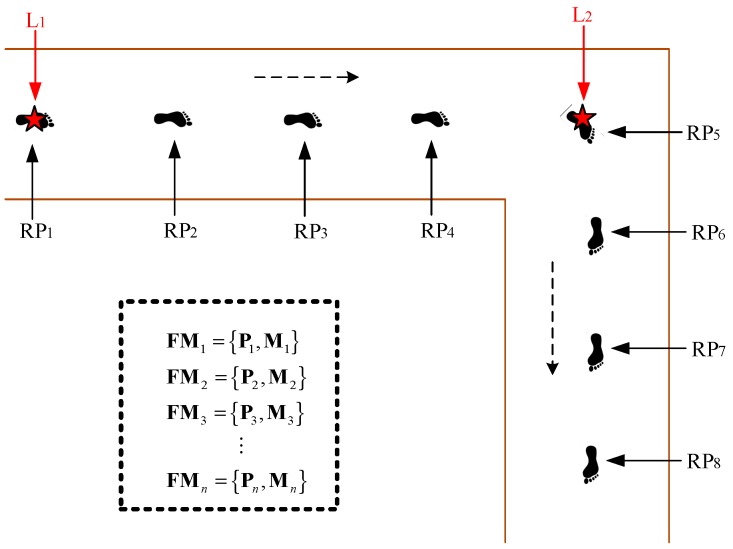
The procedure of training the magnetic map via a walking survey.

In this paper, we choose WS method to build a magnetic field map considering balancing of the mapping accuracy, efficiency and labor costs. Concretely, we first select a number of waypoints (e.g., a corner or a pillar), considering the actual spatial structure of the interested area, data collection efficiency, and data sampling density, and we get the coordinates of all the waypoints referring to the existing digital plane map. Second, the surveyors walk along a straight line at a constant speed from one waypoint to the next waypoint and mark the time when passing by each waypoint. Finally, the coordinates of the RPs between the adjacent waypoints are calculated by linear interpolation of time. Figure 3 shows the procedure of training the magnetic map using WS method. The symbol “$L_{i}$” represents a waypoint, “$RP_{i}$” represents an RP, and $FM_{i}=\left\{P_{i},M_{i}\right\}$ represents the magnetic field fingerprint of an RP. The coordinates of $RP_{i}$ between “$L_{1}$” and “$L_{2}$” can be calculated as follows:

(11) $$P_{RP_{i}}=P_{L_{1}}+\frac{T_{RP_{i}}-T_{L_{1}}}{T_{L_{2}}-T_{L_{1}}}\left(P_{L_{2}}-P_{L_{1}}\right)$$

where T represents the time of passing a waypoint.

**Figure 4:**
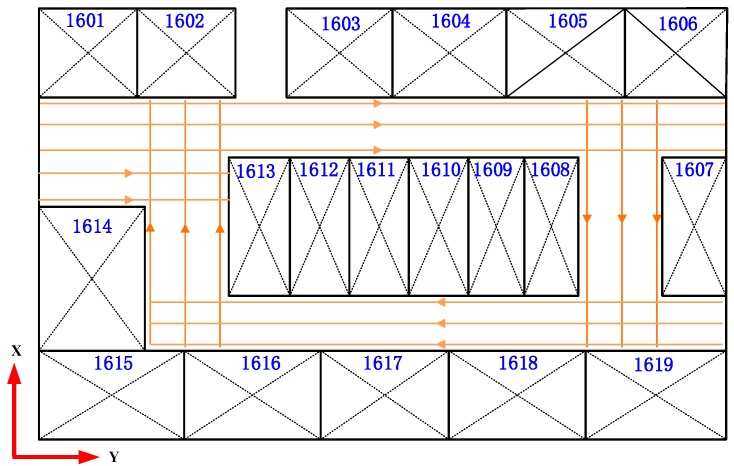
The designed trajectories for building a magnetic field map.

To generate a magnetic field map with evenly distributed RPs, we collect the magnetic field data along the lines parallel to the x-axis of the room or along lines parallel to the y-axis, as shown in Figure 4. Finally, a linear interpolation method introduced by [16] is used to produce a grid magnetic field map with a resolution of 0.1 m.

#### 2.3.2. Online Positioning Phase

Unlike for a Wi-Fi fingerprint that has high data dimensionality for matching, a maximum of three components is far from enough to achieve accurate matching positioning for a magnetic field fingerprint. Thus, we use the historical magnetic field data and corresponding relative coordinates to construct an MFS with contour features (i.e., spatial topological relationship composed of the relative coordinates), as shown in the following equation:

(12) $$Seq_{i}=\left\{\begin{array}{c} \begin{array}{cc} P_{i-k+1} & M_{i-k+1} \end{array}\\ \cdots \\ \begin{array}{cc} P_{i-1} & M_{i-1} \end{array}\\ \begin{array}{cc} P_{i} & M_{i} \end{array} \end{array}\right\}$$

where *k* represents the length of magnetic field data and *i* is the epoch of an MFS. Next, we will describe how to use the Gauss-Newton iteration method to perform magnetic field contour matching positioning. As per the description in Section 2.3.1, a grid magnetic field map is composed of many discrete RPs, which limits the precision of obtaining a reference magnetic field strength value. Thus, a bilinear interpolation is employed to obtain a more precise reference magnetic field strength value from the grid magnetic field map.

**Figure 5:**
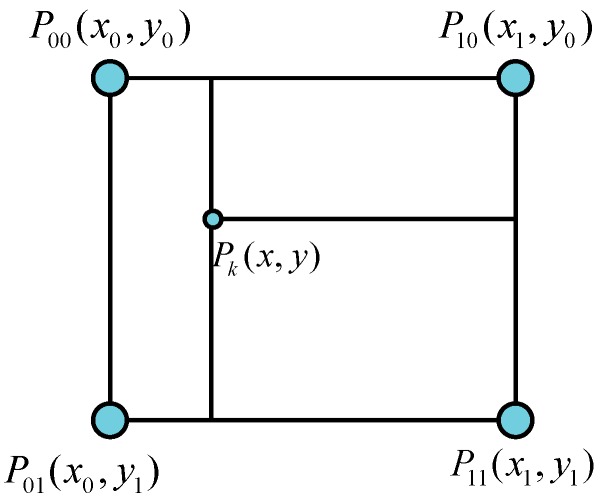
The reference magnetic field strength value from bilinear interpolation at $P_{k}$.

Given the coordinates $P_{k}$ of an observation point, the corresponding reference magnetic field strength vector $M\left(P_{k}\right)$, and the gradient vectors $\frac{\partial M\left(P_{k}\right)}{\partial x}$ and $\frac{\partial M\left(P_{k}\right)}{\partial y}$ can be approximated using the coordinates of the four closest RPs $\left(P_{00},P_{01},P_{10},P_{11}\right)$, as depicted in Figure 5. A linear interpolation along the x-axis and y-axis then yields [46]:

(13) $$\begin{array}{ll} M\left(P_{k}\right) & \approx \frac{y-y_{0}}{\left(x_{1}-x_{0}\right)\left(y_{1}-y_{0}\right)}\left[\left(x-x_{0}\right)M\left(P_{11}\right)+\left(x_{1}-x\right)M\left(P_{01}\right)\right]\\ & +\frac{y_{1}-y}{\left(x_{1}-x_{0}\right)\left(y_{1}-y_{0}\right)}\left[\left(x-x_{0}\right)M\left(P_{10}\right)+\left(x_{1}-x\right)M\left(P_{00}\right)\right] \end{array}$$

The derivatives can be expressed as follows:

(14) $$\begin{array}{ll} \frac{\partial M\left(P_{k}\right)}{\partial x} & \approx \frac{y-y_{0}}{\left(x_{1}-x_{0}\right)\left(y_{1}-y_{0}\right)}\left(M\left(P_{11}\right)-M\left(P_{01}\right)\right)\\ & +\frac{y_{1}-y}{\left(x_{1}-x_{0}\right)\left(y_{1}-y_{0}\right)}\left(M\left(P_{10}\right)-M\left(P_{00}\right)\right) \end{array}$$

(15) $$\begin{array}{ll} \frac{\partial M\left(P_{k}\right)}{\partial y} & \approx \frac{x-x_{0}}{\left(x_{1}-x_{0}\right)\left(y_{1}-y_{0}\right)}\left(M\left(P_{11}\right)-M\left(P_{10}\right)\right)\\ & +\frac{x_{1}-x}{\left(x_{1}-x_{0}\right)\left(y_{1}-y_{0}\right)}\left(M\left(P_{01}\right)-M\left(P_{00}\right)\right) \end{array}$$

The basic principle of MM is to find a rigid transformation so that the measured MFS is optimally aligned with a reference MFS coming from the magnetic field map. The proposed MM method uses the GNI algorithm inspired by the work in Lidar 2D SLAM [46] to find this rigid transformation. The GNI algorithm can help us quickly find the optimal transformation parameters based on the magnetic field gradient instead of traversing a fixed area in the magnetic field map.

We assume the rigid transformation as:

(16) $$\xi ={\left[p_{x0},p_{y0},\Delta \varphi \right]}^{T}$$

where $\left(p_{x0},p_{y0}\right)$ and Δφ represent the shift scale and rotation scale for the measured MFS, respectively. Our aim is to find a rigid transformation ξ to minimize:

(17) $$\xi^{*}=\underset{\xi }{\arg\min}\sum_{k=1}^{n}\sum_{i=1}^{2}{\left(M_{k,i}-M_{i}\left(P_{k}\left(\xi \right)\right)\right)}^{2}$$

where $M_{k,i}$ represents the measured MFS of the tri-magnetometer, $M_{i}\left(P_{k}\left(\xi \right)\right)$ is the reference MFS coming from the magnetic field map. *k* represents the measurement epoch, *i* is the element of a magnetic field fingerprint vector, and $P_{k}\left(\xi \right)$ is a function of ξ, as per the following equation:

(18) $$P_{k}\left(\xi \right)=\left(\begin{array}{cc} \cos\left(\Delta \varphi \right) & -\sin\left(\Delta \varphi \right)\\ \sin\left(\Delta \varphi \right) & \cos\left(\Delta \varphi \right) \end{array}\right)\left(\begin{array}{c} p_{x,k}\\ p_{y,k} \end{array}\right)+\left(\begin{array}{c} p_{x0}\\ p_{y0} \end{array}\right)$$

where $\left(p_{x,k},p_{y,k}\right)$ represents the estimated coordinates of the measured sequence coming from the INS mechanization. Given the starting estimated ξ, we want to estimate Δξ, which optimizes the measure error according to:

(19) $$\sum_{k=1}^{n}\sum_{i=1}^{2}{\left(M_{k,i}-M_{i}\left(P_{k}\left(\xi +\Delta \xi \right)\right)\right)}^{2}\to 0$$

Based on the first order Taylor expansion of $M_{i}\left(P_{k}\left(\xi +\Delta \xi \right)\right)$, we obtain:

(20) $$\sum_{k=1}^{n}\sum_{i=1}^{2}{\left(M_{k,i}-M_{i}\left(P_{k}\left(\xi \right)\right)-\nabla M_{i}\left(P_{k}\left(\xi \right)\right)\frac{\partial P_{k}\left(\xi \right)}{\partial \xi }\Delta \xi \right)}^{2}\to 0$$

This equation is minimized by setting the partial derivative with respect to Δξ to zero:

(21) $$2{\sum_{k=1}^{n}\sum_{i=1}^{2}\left[\nabla M_{i}\left(P_{k}\left(\xi \right)\right)\frac{\partial P_{k}\left(\xi \right)}{\partial \xi }\right]}^{T}\left[M_{k,i}-M_{i}\left(P_{k}\left(\xi \right)\right)-\nabla M_{i}\left(P_{k}\left(\xi \right)\right)\frac{\partial P_{k}\left(\xi \right)}{\partial \xi }\Delta \xi \right]=0$$

Solving for Δξ yields the Gauss-Newton equation for the minimization problem:

(22) $$\Delta \xi =H^{-1}{\sum_{k=1}^{n}\sum_{i=1}^{2}\left[\nabla M_{i}\left(P_{k}\left(\xi \right)\right)\frac{\partial P_{k}\left(\xi \right)}{\partial \xi }\right]}^{T}\left[M_{k,i}-M_{i}\left(P_{k}\left(\xi \right)\right)\right]$$

where:

(23) $$H={\left[\nabla M_{i}\left(P_{k}\left(\xi \right)\right)\frac{\partial P_{k}\left(\xi \right)}{\partial \xi }\right]}^{T}\left[\nabla M_{i}\left(P_{k}\left(\xi \right)\right)\frac{\partial P_{k}\left(\xi \right)}{\partial \xi }\right]$$

An approximation for the magnetic field gradient $\nabla M_{i}\left(P_{k}\left(\xi \right)\right)$ can be found in Equations (14) and (15). From Equation (16), we obtain:

(24) $$\frac{\partial P_{k}\left(\xi \right)}{\partial \xi }=\left(\begin{array}{ccc} 1 & 0 & -\sin\left(\Delta \varphi \right)p_{x,k}-\cos\left(\Delta \varphi \right)p_{y,k}\\ 0 & 1 & \cos\left(\Delta \varphi \right)p_{x,k}-\sin\left(\Delta \varphi \right)p_{y,k} \end{array}\right)$$

Thus, the Gauss-Newton Equation (19) can now be evaluated, yielding a step closer to the minimum. It is important to note that the algorithm works on non-smooth linear approximations of the magnetic field gradient, meaning that the local quadratic convergence towards a minimum cannot be guaranteed. Nevertheless, the algorithm works with sufficient accuracy in practice.

### 2.4. INS/MM Integrated Navigation Solution

INS-based navigation is a relative positioning method, and its position errors are proportional to the distance traveled, and the magnetic field contour matching positioning is greatly affected by trajectory accuracy. To further improve the navigation performance, the positioning result of MM is also used to control the position drift of the INS-based PDR in the integrated system. The misclosure of the positioning result of MM in the n-frame is given by:

(25) $$\delta z_{p}={\hat{p}}_{INS}^{n}-{\tilde{p}}_{MM}^{n}$$

where ${\hat{p}}_{INS}^{n}$ represents the position vector calculated by INS in the *n*-frame, and ${\tilde{p}}_{MM}^{n}$ is the position error vector of MM. The observation equation is formulated as:

(26) $$\delta z_{r}=H_{MM}\delta x+n_{MM}$$

where $n_{MM}$ represents the white noise of the MM position and $H_{MM}$, the corresponding design matrix, can be expressed as:

(27) $$H_{MM}=[\begin{array}{cc} I_{3\times 3} & 0_{3\times 12} \end{array}]$$

## 3. Results

**Figure 6:**
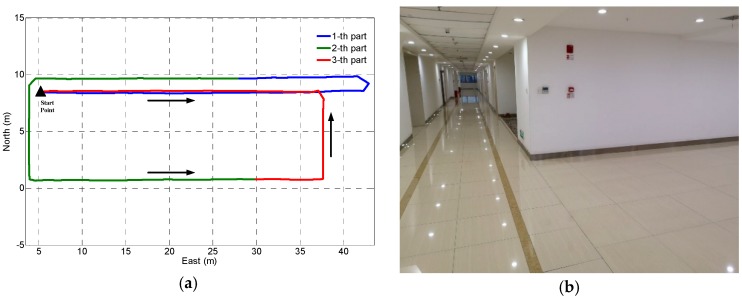

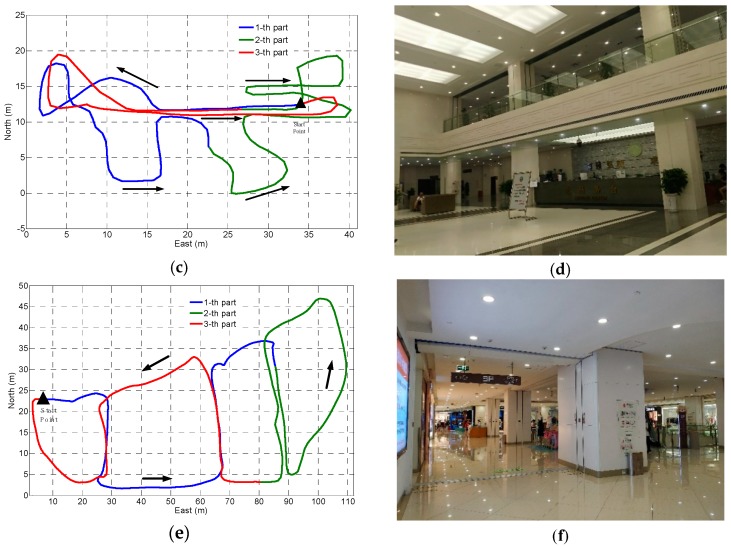
Trajectories of the experiments. (**a**) The trajectory in building A; (**b**) experimental environment of building A; (**c**) the trajectory in building B; (**d**) experimental environment of building B (**e**) the trajectory in building C; (**f**) experimental environment of building C.

To evaluate the performance of the proposed indoor positioning algorithm, several experiments were performed by three pedestrians following two different trajectories. As shown in Figure 6, the first trajectory is in building A (approximately 300 m^2^), which is a typical indoor office building environment with a narrow corridor. The second trajectory is in building B (approximately 800 m^2^), which is a typical lobby in an indoor environment. The third trajectory is in building C (approximately 3800 m^2^), which is a typical shopping mall indoor environment. Two different smartphones (e.g., Google Pixel 2 and Huawei P10) with built-in tri-gyroscopes, tri-accelerometers, and tri-magnetometers were used to collect the experimental data. To eliminate the effects of hard iron, the bias calibration process of the tri-magnetometers was implemented before experimental data collection and navigation performance evaluation. An Android app developed by the authors is used for data collection in the sensors calibration phase, offline training phase and online positioning phase.

In the online positioning phase, a participant hold a smartphone in hand and keep the smartphone about 1.2 m from the ground. There is no significant position change between the smartphone and the participant’s body and no large angle difference (e.g., less than 10 degree) between the heading of the smartphone and the walking direction of the participant.

### 3.1. Step Detection Failure for INS-Based PDR

In this section, we evaluate the influence of step detection failure for INS-based DPR. We know that any step detection failure will cause a significant damage to the navigation performance of the gait-based PDR. But the INS-based PDR has the ability to maintain its navigation performance when the step detection failure only occurs for several seconds.

**Figure 7:**
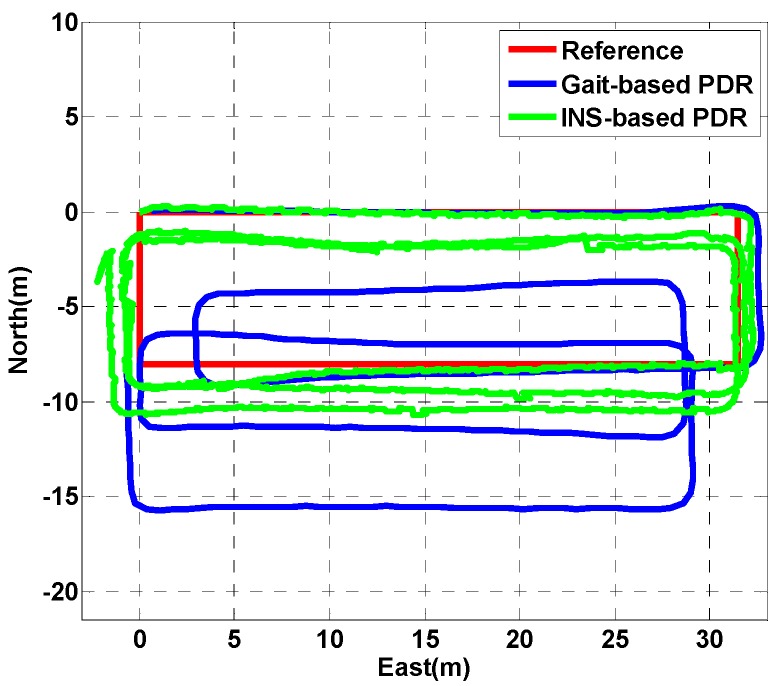
Trajectories of reference, gait-based PDR and INS-based PDR.

We set up an experiment in which the pedestrian walks a total of 340 steps and randomly five steps detection failure (about 3 s) for a total of eight times. Figure 7 shows the position estimation performance of gait-based PDR and INS-based PDR in the presence of step detection failure. In Figure 7, INS-based PDR exhibits a slight decrease in position accuracy when missing a small number of steps. However, the position result calculated by gait-based PDR is highly sensitive the step detection failure. According to the average step length of 0.65 m, five steps will bring a position error of 3.25 m to the gait-based PDR.

### 3.2. Magnetic Field Map

**Figure 8:**
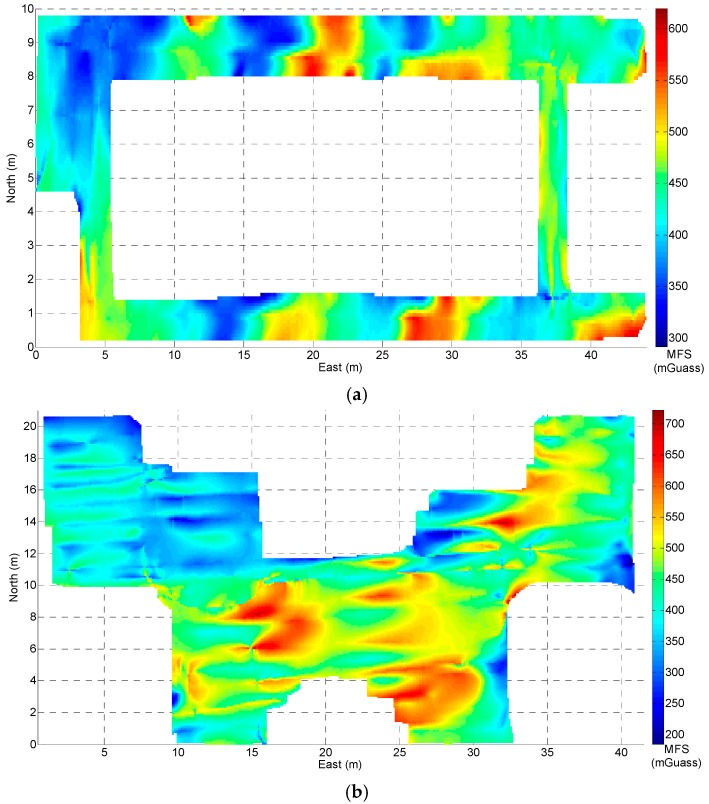

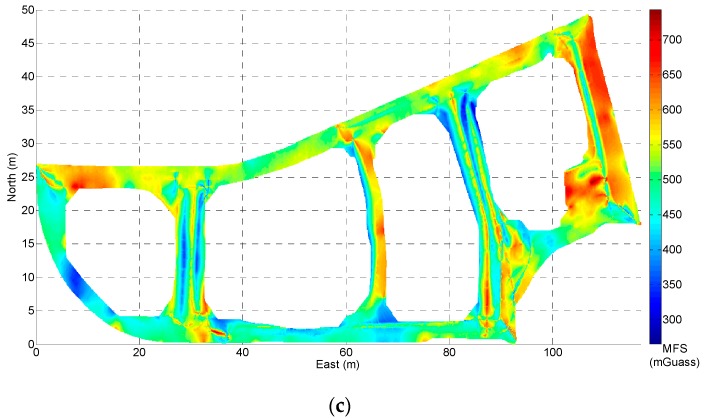
The magnetic field strength map. (**a**) Building A; (**b**) building B; (**c**) building C.

After data collection, a low-pass filter was used to reduce the effects of sensor noise and human motion, and the linear interpolation method described above was used to generate the magnetic field map, as shown in Figure 8. From the magnetic field map of building A, B and C, although the magnetic field data acquisition followed many sparse lines, the high-frequency information of the magnetic field fingerprint was still retained.

We can see that the magnetic field strength is the same in multiple places, which can easily lead to the mismatching of a single point in the magnetic field fingerprint based on the matching positioning. Fortunately, there is a sharp fluctuation of the indoor magnetic field strength in a small area. Based on this phenomenon, we constructed a new magnetic field feature by using the magnetic field sequence combined with the trajectory in order to achieve better magnetic field matching. As Figure 8 shows, the variation in the magnetic field strength at a distance of 0.1 m is small and can be ignored. 

The smartphone’s built-in tri-magnetometer always has a high sampling rate (e.g., more than 20 Hz), and the normal walking velocity of a pedestrian is usually approximately 1.2 m/s. Therefore, it is necessary to generate a sparse MFS (i.e., one sampling point at intervals of 0.1 m) by resampling the collected magnetic field data for a low computational cost on the online positioning phase. In this paper, we use an MFS with a length of 7 m to perform the position estimation.

### 3.3. Position Estimation Performance Analysis

In this section, we analyze the navigation performance of the INS (i.e., the INS-based navigation as described in Section 2.2), MM and MM/INS integrated method. MM and MM/INS integrated method are both using the trajectory contour coming from INS-based PDR to improve the distinguishing of a MFS, and MM/INS integrated method will use the integrated navigation solution described in Section 2.4 compare with MM.

**Figure 9:**
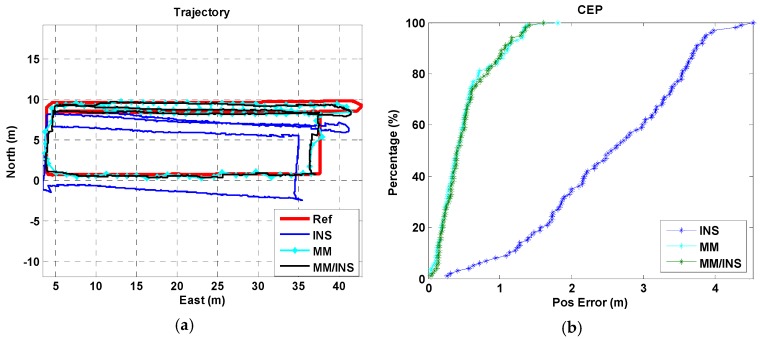
Navigation results of the INS, MM, and MM/INS integrated solutions in building A (Test I: Pedestrian I, Trajectory I, Google Pixel 2). (**a**) Trajectories; (**b**) cumulative error percentages.

**Figure 10:**
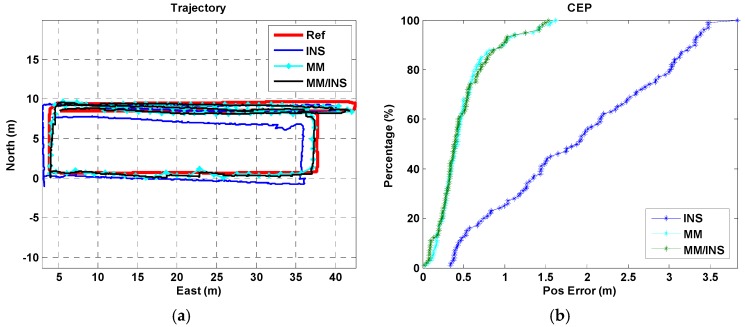
Navigation results of the INS, MM, and MM/INS solutions in building A. (Test II: Pedestrian II, Trajectory I, Huawei P10). (**a**) Trajectories; (**b**) cumulative error percentages.

**Figure 11:**
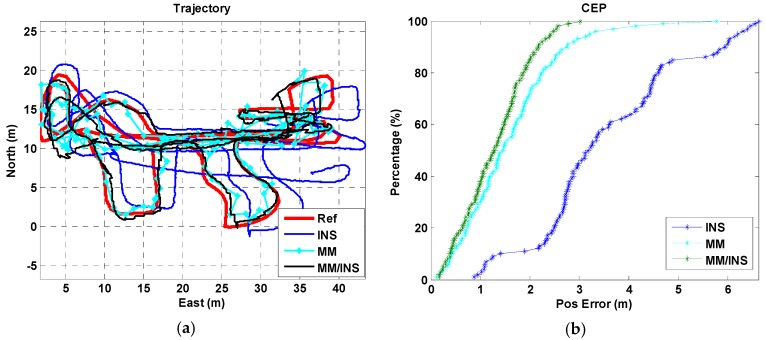
Navigation results of the INS, MM, and MM/INS solutions in building B (Test III: Pedestrian III, Trajectory II, Google Pixel 2). (**a**) Trajectories; (**b**) cumulative error percentages.

**Figure 12:**
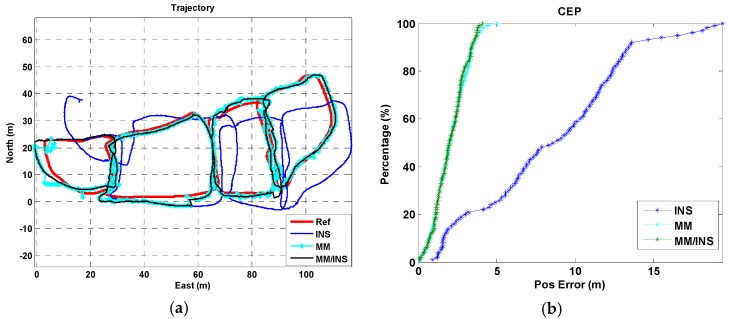
Navigation results of the INS, MM, and MM/INS solutions in building C (Test IV: Pedestrian III, Trajectory III, Google Pixel 2). (**a**) Trajectories; (**b**) cumulative error percentages.

We present a total of four tests, each of which were configured differently for the used phone and the tested trajectory. A “Google Pixel” smartphone was used in trajectory I for Test I, a “Huawei P10” smartphone was used in trajectory I for Test II, a “Google Pixel” smartphone was used in trajectory II for Test III, and a “Google Pixel” smartphone was used in trajectory III for Test IV. The position estimation results and cumulative error percentages (CEPs) of the INS, MM, and MM/INS integrated solutions are presented in Figure 9, Figure 10, Figure 11 and Figure 12. In the trajectory figures, the red, blue, cyan, and black solid lines represent the reference trajectory, position estimation of the INS, MM, and MM/INS, respectively. For the CEP figures, the blue, cyan, black lines represent the CEP of INS, MM, and MM/INS, respectively.

As Figure 9 and Figure 10 show, the proposed MM algorithm can achieve a high precision position estimation in the corridor environment. And the position estimation performance is still consistent when 2 participants hold 2 different smartphones. Figure 11 shows the position estimation performance of the proposed MM algorithm when the participant walks freely in a complex manner in the lobby environment. We can also learn that the proposed method has the ability to meet large-scale indoor positioning requirements for its good positioning estimation performance, as shown in Figure 12.

The position errors of the INS, MM, MM/INS integrated methods for the four tests are illustrated in Table 1. The MEMS–IMU-based INS solution has a root mean square (RMS) position error of 2.75 m, 2.14 m, 3.81 m and 9.59 m for the four tests, respectively. The proposed MM solution based on the magnetic field fingerprint has an RMS position error of 0.64 m, 0.60 m, 1.87 m and 2.34 m for the four tests and a matching success rate of 94.69%, 95.57%, 90.24% and 88.01% for the four tests, respectively. The “matching success rate” represents the total number of successful matches divided by the total number of matches. The MM/INS solution has an RMS position error of 0.64 m, 0.60 m, 1.45 m and 2.27 m for the four tests, respectively.

The proposed MM algorithm has an excellent position estimation performance and high matching success rate from the results of Test I and Test II in building A. We know that the estimated trajectory coming from the INS-based navigation method has the ability to improve the distinguishability of the magnetic field fingerprint. Compared with the results of Test I and Test II, Test III and Test IV had a worse navigation performance in the position estimation and matching success rate because the frequent turning movements reduced the accuracy of the step model, decreasing the accuracy of the estimated trajectory. And the underlying magnetic field of building B and building C have a gentler gradient than that of building A.

We also found that the MM/INS integrated solution does not have higher positioning accuracy than MM in the four tests. The reason is that the positioning accuracy of the MM/INS integrated navigation scheme is determined by MM, and the INS can provide only a continuous position estimation and enhance the system’s ability to resist gross errors.

Table 2 summarizes the current algorithm presented in other papers which used magnetic field fingerprint-based matching method and inertial sensors. In Table 2, we compare the results shown in other papers with similar test setup against results achieved in this paper. It shows that the proposed algorithm has better positioning accuracy when compare with the DTW based research [8,10,22,47,48]. Though the study in [22] has reported its positioning accuracy of 2 m in the corridor environment, the positioning accuracy of our proposed algorithm in the similar test scenario is 0.6 m. We also notice that the proposed algorithm has slightly worse positioning accuracy than the PF-based research [17,24,27,49]. However, the studies of [24,49] only reported the positioning accuracy in a simple magnetic field environment (e.g., corridor and bookshelves in the library), which cannot represent their positioning accuracy in a more complex environment. The research reported in [17,27] had a very excellent positioning performance in typical indoor environments (e.g., hall, conference room, corridor and bookshelves in the library), but it will cause a lot of power consumption of the smartphone for its large computational load.

## 4. Discussion

As described in Section 3, the proposed algorithm has significant differences in its positioning performance in the three buildings because the accuracy of the magnetic fingerprint-based positioning method is highly determined by the distribution of the underlying magnetic field. The experimental results show that the proposed algorithm provides similar positioning accuracy compared to the existing algorithms.

However, the advantages of the proposed algorithm are still obvious. Compared with the DTW algorithm [8,10,13,20,22,48,50,51], the proposed algorithm uses a grid magnetic field map, so the size of the magnetic field map is only related to the size of the actual environment rather than the number of predicted trajectories. More importantly, the DTW algorithm must be able to predict the user’s trajectory well on the training phase for achieving better positioning performance, which is essentially a one-dimensional matching algorithm. The proposed algorithm has a more excellent performance in positioning accuracy and robustness for the Insensitivity to user walking trajectories and the higher distinguishability of a magnetic field fingerprint combine with the trajectory contour (from PDR). The particle filter [16,17,23,24,25,26,27,28,29,30] overcomes the shortcomings of the DTW algorithm, and is a promising magnetic fingerprint algorithm that provides high precision and high stability, but it is still computationally intensive for a smart phone. The Gauss-Newton iterative method converges along the direction of the fastest drop of the magnetic field gradient, and has the characteristics of low computational complexity and fast convergence. So the proposed algorithm is more suitable for smartphones because of the lightweight computational load.

The proposed magnetic matching algorithm has the following limitations that needs to be handled in the future. Due to the initial navigation state (i.e., initial position and initial heading) plays an important role in the proposed MM method, it rely on other methods to do navigation state initialization [52]. In addition, the user poses of using smartphones vary widely, and it is a very challenging task to use MEMS-IMU to provide reliable high-precision relative trajectories.

## 5. Conclusions

In this paper, we present an ambient magnetic field-based positioning method for smartphones in an indoor environment. To improve the position estimation accuracy of magnetic field matching positioning, we analyze the underlying magnetic field of three typical indoor environments (i.e., a corridor, an open area and a shopping mall). Then, we use the magnetic field sequence combined with the estimated trajectory coming from the INS-based PDR, which improve the distinguishability of the magnetic field fingerprint and design a Gauss-Newton iteration method to perform magnetic field contour matching positioning. To evaluate the navigation performance of the proposed MM algorithm, we conduct experiments in the three typical indoor environments. The test results show that the position estimation error of MM is 0.64 m (RMS) in the office building environment, 1.87 m (RMS) in a typical lobby environment and 2.34 m (RMS) in a shopping mall environment.

In our future works, we will use the WiFi-based distance intersection method to determine a small area and then the initial position is determined by traversing the entire area, and use the tri-magnetometer to calculate the initial heading. Furthermore, in order to adapt to the user’s behavior, we will test the positioning performance of the proposed MM method in the four typical smartphone poses, i.e., handheld, calling, swinging in the hand, and in pants pocket.

## Figures and Tables

**Table 1:** Position errors of the INS, MM, MM/INS integrated methods for the four tests (units of m).

	Test I			Test II			Test III			Test IV		
	Max	Mean	RMS	Max	Mean	RMS	Max	Mean	RMS	Max	Mean	RMS
**INS**	4.52	2.55	**2.75**	3.83	1.88	**2.14**	6.62	3.52	**3.81**	19.34	8.39	**9.59**
**MM**	1.80	0.50	**0.64**	1.62	0.49	**0.60**	5.77	1.59	**1.87**	5.05	2.10	**2.34**
**MM/INS Integrated**	1.61	0.52	**0.64**	1.52	0.49	**0.60**	3.03	1.29	**1.45**	4.11	2.06	**2.27**

**Table 2:** Performance comparison of the proposed method with the current methods.

Scheme	Techniques	Test Scenarios	Positioning Accuracy	Computational Load
[47]	DTW/Bayesian	Shopping mall	<5 m	Medium
[8]	DTW, Wi-Fi	Corridor	4 m	Low
[48]	DTW	Corridor, UPL, Supermarket	<4 m	High
[10]	DTW	Corridor	3.5 m	Low
The Proposed Scheme	GN	Corridor, Hall, Shopping mall	<2.5 m	Low
[22]	DTW	Corridor	<2 m	Medium
[17,27]	PF	Hall, Conference Room, Corridor, Book shelf	<2 m	Medium
[49]	PF	Corridor	1 m	High
[24]	PF, IM	Book shelf	0.75 m	Medium

DTW—Dynamic Time Warp, PF—Particle Filter, GN—Gauss-Newton, HMM–Hidden Markov Model, IM—Indoor Map, UPL—Underground Parking Lot.
